# Supplementary material for: The Presence of Human Herpesvirus 6 in the Brain in Health and Disease
Source: Biomolecules. 2020 Nov 6;10(11):1520. doi: 10.3390/biom10111520 (PMC7694807; doi:10.3390/biom10111520)
Supplement: Supplementary file 1 [file biomolecules-10-01520-s001.zip › biomolecules-985854-supplementary/Table_1_formatted.docx]

| **Protein target** | **Stage** | **Localization** | **Antibody** | **Type** | **Species** | **Source** | **Ref** | **Cell type** |
| --- | --- | --- | --- | --- | --- | --- | --- | --- |
| p101 | Late | Tegument | **C3108-103** | Mouse  monoclonal | B | Dr. Philip Pellet (Pellet et al. 1993) | Drobyski et al. 1994 Knox et al. 1995 Challoner et al. 1995 Saito et al. 1995 Friedman et al. 1999 | Astrocytes  Neurons  Oligodendrocytes  Macrophague  Microglia |
|  |  |  |  |  |  | Chemicon | Challoner et al. 1995 Friedman et al. 1999 Mock et al. 1999 Ito et al. 2000 Blumberg et al. 2000 Wainwright et al. 2001 Goodman et al. 2003 | Astrocytes  Neurons  Oligodendrocytes  Microglia Endothelial Lymphocytes |
|  |  |  |  |  |  | USBiological Cat# H2034-17A | Opsahl et al. 2005 | Oligodendrocytes |
|  |  |  |  |  |  | Abcam Cat# ab64536 | Li et al. 2011 | Astrocytes Microglia |
|  |  |  |  |  |  | Abcam Cat# ab128404 | Liu et al. 2018 | Astrocytes Neurons |
|  |  |  | Unk | Unk |  | Biometria | Novoa et al . 1997 | Astrocytes Oligodendrocytes Lymphocytes |
|  |  |  | Unk | Unk |  | Virotech | Wang et al. 1999 | Microglia Macrophages Endothelial Lymphocytes |
| Unk | Unk | Unk | #30-HSB | Rabbit antiserum | Unk | Dr. Donald Carrigan (Russler et al. 1991) | Drobyski et al. 1994 Knox et al. 1995 Mckenzie et al. 1995 Carrigan et al. 1996 | Astrocytes  Neurons  Oligodendrocytes  Microglia |
| p41 | Early | Nuclear | C5 | Mouse monoclonal | A and B | Biodesign (Agulnick et al. 1993) | Challoner et al. 1995 | Astrocytes  Neurons  Oligodendrocytes  Macrophague |
|  |  |  | Unk | Unk | Unk | Virotech | Mock et al. 1999 Ito et al. 2000 Blumberg et al. 2000 Wainwright et al. 2001 Goodman et al. 2003 | Neurons Oligodendrocytes Microglia Endothelial Lymphocytes |
|  |  |  | 9A5D12 | Mouse monoclonal | A and B | Dr. Bala Chandran (Balachandran et al. 1989) | Saito et al. 1995  Wagner et al. 1997 | N/A |
|  |  |  | Unk | Mouse monoclonal | Unk | Autogen-Bioclear | Opsahl et al. 2005 | Oligodendrocytes |
|  |  |  | Unk | Unk | Unk | Unk | Cuomo et al. 2001 | Glial cells (Schwann) |
| gp116/gp64/gp54 | Late | Core | 6A5D5 | Mouse monoclonal | A and B | Dr. Bala Chandran | Saito et al. 1995 | N/A |
|  |  |  | 6A6G3 | Mouse monoclonal | A and B | Advanced Biotechnologies Cat# 13-219-001 or 13-218-100 (Balachandran et al. 1989) | Goodman et al. 2003 Donati et al. 2003 Fotheringham et al. 2007a Fotheringham et al. 2007b Niehusmann et al. 2010 Esposito et al. 2015 | Astrocytes  Neurons  Oligodendrocytes  Macrophages Lymphocytes |
|  |  |  |  |  |  | HHV-6 Foundation | Huang et al. 2015 | Neurons |
|  |  |  | Unk | Mouse monoclonal | A and B | Autogen-Bioclear | Opsahl et al. 2005 | Oligodendrocytes |
| gp82/gp105 | Late | Envelope | UK82 | Rabbit antiserum | Unk | Dr. Bala Chandran | Saito et al. 1995 | N/A |
|  |  |  | 2D6 | Mouse monoclonal | A | Dr. Bala Chandran (Balachandran et al. 1989) | Knox et al. 2000 | N/A |
| gp110/60 | Late | Envelope | H-AR3 | Mouse monoclonal | Unk | Dr. Luca | Wagner et al. 1997 | Astrocytes  Neurons  Oligodendrocytes |
|  |  |  | Unk | Unk | Unk | Unk | Le Guennec et al. 2017 | Glial cells |
| gH | Late | Envelope | OHV-3 | Mouse monoclonal | B | Advanced Biotechnologies (Okuno et al. 1990) | Knox et al. 2000 | N/A |
|  |  |  |  |  |  | HHV-6 Foundation | Huang et al. 2015 | Neurons |
| 37KDa early antigen | Early | Unk | 1.B.367 | Mouse monoclonal | A | USBiological Cat# H2034-01 | Opsahl et al. 2005 | Oligodendrocytes |
| U94 | Latency | Nuclear | MORI | Mouse monoclonal | B | HHV-6 Foundation | Huang et al. 2015 | Neurons |
| Unk | Unk | Unk | sc-65463 | Mouse monoclonal | A and B | Santa Cruz | Chapenko et al. 2016 Skuja et al. 2017 | Astrocytes  Oligodendrocytes  Microglia Endothelial Lymphocytes Fibroblasts |
